# Elevated mitochondrial DNA copy number in peripheral blood cells is associated with childhood autism

**DOI:** 10.1186/s12888-015-0432-y

**Published:** 2015-03-17

**Authors:** Shan Chen, Zongchang Li, Ying He, Fengyu Zhang, Hong Li, Yanhui Liao, Zhen Wei, Guobin Wan, Xi Xiang, Maolin Hu, Kun Xia, Xiaogang Chen, Jinsong Tang

**Affiliations:** The Second Affiliated Hospital and Yuying Children’s Hospital of Wenzhou Medical University, Wenzhou, 325027 China; Institute of Mental Health of the Second Xiangya Hospital, National Laboratory for Psychiatric Disease Diagnosis and Treatment, Key Laboratory of Psychiatry and Mental Health of Hunan Province, The Central South University, Changsha, China; The National Clinical Research Center for Psychiatric and Psychological Diseases, Changsha, China; Institute of Genomic Medicine, Wenzhou Medical University, Wenzhou, Zhejiang China; Division of Clinical Sciences, Lieber Institute for Brain Development, John Hopkins University Medical Campus, 855 N. Wolfe Street, Suite 300, Baltimore, 21205 MD USA; Department of Women’s Health Care, The Affiliated Shenzhen Maternal and Child Health Care Hospital, Nanfang University of Medical Science, Shenzhen, China; BGI Ark Biotechnology Co., Ltd., Shenzhen, Guangdong China; The State Key Laboratory of Medical Genetics, Central South University, Changsha, Hunan China

**Keywords:** Autism, Mitochondrial dysfunction, mtDNA copy number, CARS, ABC

## Abstract

**Background:**

Several lines of evidence indicate mitochondrial impairment in the pathophysiology of autism. As one of the most common biomarkers for mitochondrial dysfunction, mitochondrial DNA (mtDNA) copy number has also been linked to autism, but the relationship between mtDNA copy number and autism was still obscured. In this study, we performed a case–control study to investigate whether mtDNA copy number in peripheral blood cells is related to patients with autism.

**Methods:**

Relative mtDNA copy number in peripheral blood cells was measured by using real-time polymerase chain reaction method. The participants in this study included 78 patients with childhood autism and 83 typically developing children.

**Results:**

We observed children with autism had significantly elevated relative mtDNA copy number than healthy controls (Beta = −0.173, P = 0.0003). However, there were no significant correlations between mtDNA copy number and clinical features (paternal age, maternal age, age of onset, illness of duration, CARS score and ABC score) in childhood autism.

**Conclusion:**

We show that elevated mtDNA copy number in peripheral blood is associated with autism, indicating that there may be mitochondrial dysfunction in children with autism.

## Background

Autism is a neurodevelopmental disorder, characterized by social deficits, communication impairment and unusually restricted, repetitive behaviors with onset prior to three years of age. The worldwide prevalence of autism spectrum disorder (ASD) is estimated at about 0.7%, although the estimates vary with populations [1,2]. Because of the complex, multifactorial etiology and pathophysiology underlying this illness and the limited scientific advance, the pathogenesis of autism is still elusive. It is consensually believed that gene–environment interplay contributes to the development of autism [3,4]. Increasing evidence has shown that mitochondrial dysfunction is associated with autism and there is much higher prevalence of mitochondrial diseases in autism than that in general population of children [5-7].

Mitochondria are specialized cellular organelle generating adenosine triphosphate (ATP) through oxidative phosphorylation that is a series of chemical reactions in the electron transport chain (ETC). In addition, mitochondria play important roles in other biological activities of cells, such as apoptosis and calcium regulation, and are the major intracellular source and primary target of reactive oxygen species (ROS) that is toxic for mitochondria [8,9]. In normal cells, each mitochondrion carries about 2 to 10 copies of mitochondrial DNA (mtDNA). The mtDNA is a 16.5 kb circular double-stranded molecule containing 37 genes, which codes partial proteins of ECT enzyme complexes and partial components of the machinery of intramitochondrial protein synthesis. Comparing with nuclear DNA, mtDNA is lack of protective histones and has limited DNA repair capacity, and therefore is highly susceptible to intramitochondrial ROS [10].

Increased oxidative stress has been found in patients with autism [11-13]. This leads to high rates of mutation and deletion for mtDNA, and subsequently impairs the mitochondrial function [14]. Evidence of different lines supports a role of mitochondrial impairment in the pathophysiology of autism [5,15-21]. Studies of peripheral blood and lymphoblastoid cell lines have identified ETC complexes deficiency and disordered mitochondrial energy metabolism such as plasma lactate, pyruvate, carnitine and amino acids in individuals with autism [19]. Similarly, previous magnetic resonance spectroscopy study has showed increased lactate doublets, decreased synthesis of ATP and a disturbed energy metabolism in the brain of autism [21]. Postmortem human brain studies in autism have also found decreased protein expressions of ETC complexes in specific region of brain, reinforcing the mitochondrial dysfunction in this illness [20]. Abnormal mtDNA number, as one of the most common biomarkers, has been observed associated with mitochondrial dysfunction and increased oxidative stress [22-25].

Previous studies have suggested that mtDNA copy number may increase with mtDNA damage or mitochondrial dysfunction, and compensate for the mitochondrial energy metabolism in patients with ASD [20]. Other studies failed to find this abnormality in ASD [19,26]. Considering the ASD defined by a constellation of different classes of subtypes, the diversity of ASD and the limited sample size in these studies may account for the inconsistent results. In an attempt to elucidate this association, we performed a large case–control study to investigate the status of peripheral blood mtDNA copy number in subjects with childhood autism.

## Methods

### Subjects

The study included 78 children with autism and 83 typically developing children. All subjects age from 3 to 6 years and were Han Chinese ethnicity. Children with autism were consecutively recruited from the department of Children psychology, MCH Hospital of Shenzhen and Institute of Mental Health, Second Xiangya Hospital of Central South University from July 2011 to December 2012. All patients were diagnosed by two senior psychiatrists according to DSM-IV diagnostic criteria-based structured interview for autism. In addition, all cases were also assessed by using childhood autism rating scale (CARS) and autism behavior checklist (ABC) [27,28]. Exclusion criteria for children included: autistic patients with age above 6 years, patients with Rett syndrome, childhood schizophrenia, Asperger’s syndrome or pervasive developmental disorder-not otherwise specified (PDD-NOS).

All typically developing children were recruited from community volunteers. The current mental status and history of mental disorders of the control subjects were evaluated by a senior psychiatrist. Controls recruited when individuals met the criteria and did not have any history of mental disorders, neurological disorders, substance abuse and serious physical diseases. Unfortunately we failed to obtain a family history of mental disorders.

Written informed consents conforming to the principles expressed in the Declaration of Helsinki were obtained from the guardians of all participants. The study was approved by the Human Ethics Committee of the Second Xiangya Hospital.

### Clinical assessment

The severity of autism was assessed by CARS, which rates the child on a scale from one to four in each of 15-items behavioral rating scale. The items are: relating to people; emotional response; imitation; body use; object use; listening response; fear or nervousness; verbal communication; non-verbal communication; activity level; level and consistency of intellectual response; adaptation to change; visual response; taste, smell and touch response and general impressions. Children with a total score above 30 were considered autistic and were included in this study [27].

The ABC consists in 57 items about the atypical behaviors and these behaviors are related to five areas (sensory stimuli sensorial; relating; body and object use; language social; self-help). Most items scored from 1 to 4 according to the impairment degree. Children with scores above 53 were considered autism and were included in this study [28].

### Laboratory analysis

Peripheral blood samples were collected in EDTA tubes and kept at −20°C before use. Genomic DNA was isolated from 200 ul of each blood sample using a commercial DNA Isolation Kit NEP004-1 (Beijing Dingguo Changsheng Biotechnology Co., Ltd., China). The procedure for DNA extraction and purification was performed by using the silica-membrane-based spin column method. The quantity and purity of the DNA was assayed using a Nanodrop 2000 spectrophotometer (Thermo Scientific, Wilmington, DE, USA) and all DNA samples all had OD260/OD280 values of 1.7–2.0. Then total DNA samples were stored at −70°C until use.

The relative mtDNA copy number was measured by quantitative real-time polymerase chain reaction (PCR) and normalized by simultaneous measurement of the nuclear DNA according the method described in previous studies [29-31]. In brief, the primer sequences L394, 5′-CACCAGCCTAACCAGATTTC-3′/H475, 5′-GGGTTGTATT-GATGAGATTAGT-3′ were used for measuring the mtDNA content, and primers HBG1F, 5′-GCTTCTGACACAACTGTGTTCACTAGC-3′/HBG1R 5′-CACCAACTTCATCCACGTTCACC-3′ were used for amplification the single-copy nuclear ß-globin gene [29]. Assay was performed by using the Maxima SYBR Green qPCR Master Mix (Thermo Fisher Scientific, Waltham, MA, USA) supplied by CFX96 Real-Time Detection Systems (BioRad Laboratories, Hercules, CA, USA). The qPCR was performed under the following conditions: denaturation at 95°C for 10 minutes followed by 40 cycles of 10s at 95°C, 30 at 60°C and 30s at 72°C. All assays were carried out in triplicate using 10 ng DNA per 10 μl reaction. The acceptable standard deviation (SD) of the triplicate threshold cycle (Ct) values was set at 0.3. If the result was out of the acceptable range, then the run was repeated for the same sample. The relative mtDNA copy number was calculated by the equation 2^−ΔΔCt^ (ΔCt = Ct_mtDNA_-Ct_β_-globin) [32,33].

### Statistical analysis

All statistical analyses were performed with the Statistical Package for the Social Sciences (SPSS, version 20.0 for Windows). The difference in the distribution of characteristics between the cases and the controls was evaluated by chi-square test for categorical variables (gender) and Student’s t-test for continuous variables (age). Age and sex of the participants, as well as the batch used for mtDNA copy number measurement, were included as covariates in linear regression in order to rule out confounding effects. For comparisons between multiple groups, one-way ANOVA followed by LSD test for multiple comparisons between any of the two groups was carried out. Monotonic relationships between mtDNA copy number and clinic parameters of autism were examined by using linear regression analysis. All P-values were two-sided and considered statistically significant at <0.05.

## Results

A total 78 patients with autism and 83 healthy controls were included in this study. The mean age of autism patients and healthy controls were 45.4 ± 12.3 and 47.3 ± 12.5 months, respectively (p = 0.413, Table 1). There was no sex difference between autism cases and healthy control samples (p = 0.175), although there were more female controls (n = 16) than cases (n = 9). In addition, the mean CARS and ABC scores in cases were 34.48 ± 3.01 and 105.44 ± 9.55, respectively. The relative mtDNA copy number was skewed distributed in both autism cases and healthy control. The mean of relative mtDNA copy numbers was 9.16 (SD = 11.6) in cases and 3.052 (SD = 4.252) in controls; both were far from its medians, 1.624 for autism cases and 1.257 for controls (Table 2). There was a quite large standard deviation in both groups. We performed eighth root transformation, which made the means and median of transformed mtDNA very close in both autism cases (1.135 ± 0.293 vs 1.062) and controls (0.960 ±0.288 vs 1.029), suggesting the transformed mtDNA are symmetric distribution, and variations were close in two groups.

**Table 1:** **Demographic and clinical characteristics of autism patients and healthycontrols**

**Variables**	**Cases (n = 78)**	**Controls (n = 83)**	**P value**
Age, months (mean±SD)	45.4±12.3	47.3±12. 5	0.413
Sex, n, (%)			0.175
Male	69 (88.46)	67 (80.72)	
Female	9 (11.54)	16 (19.23)	
Family training (yes/no)*	27/35		
Paternal age, years (mean±SD)*	30.18±4.64		
Maternal age, years (mean±SD)*	27.34±4.68		
Age of onset, years (mean±SD)*	2.05±0.71		
Illness of duration, years (mean±SD)*	2.49±1.20		
CARS Score, (mean±SD)*	34.48±3.01		
ABC Score, (mean±SD)*	105.44±9.55		

*Data from 16 of 78 cases (same subjects) were missing.

**Table 2:** **Summary statistics of relative mtDNA in autism cases and controls and by sexin autism cases and controls**

	**Status**	**N**	**Variable**	**Mean**	**Median**	**SD**	**Min**	**Max**	**25th Pctl**	**75th Pctl**
Overall										
	Control	83	mtDNA	3.052	1.257	4.252	0.004	23.702	0.041	5.153
			sqrtmtDNA	0.960	1.029	0.288	0.495	1.485	0.671	1.227
	Autism	78	mtDNA	9.165	1.624	11.596	0.015	35.585	0.807	19.004
			sqrtmtDNA	1.135	1.062	0.293	0.590	1.563	0.974	1.445
Male										
	Control	67	mtDNA	3.056	0.979	4.454	0.004	23.702	0.045	4.885
			sqrtmtDNA	0.955	0.997	0.289	0.495	1.485	0.679	1.219
	Autism	69	mtDNA	8.994	1.617	11.369	0.015	35.585	0.762	18.724
			sqrtmtDNA	1.131	1.062	0.295	0.590	1.563	0.967	1.442
Female										
	Control	16	mtDNA	3.037	1.585	3.399	0.014	8.473	0.036	6.681
			sqrtmtDNA	0.979	1.057	0.291	0.589	1.306	0.660	1.267
	Autism	9	mtDNA	10.478	2.570	13.908	0.038	32.939	1.030	20.978
			sqrtmtDNA	1.167	1.125	0.298	0.665	1.548	1.004	1.463

Note: sqrtmtDNA—Eighth-root transformation of mtDNA.

**Figure 1:**
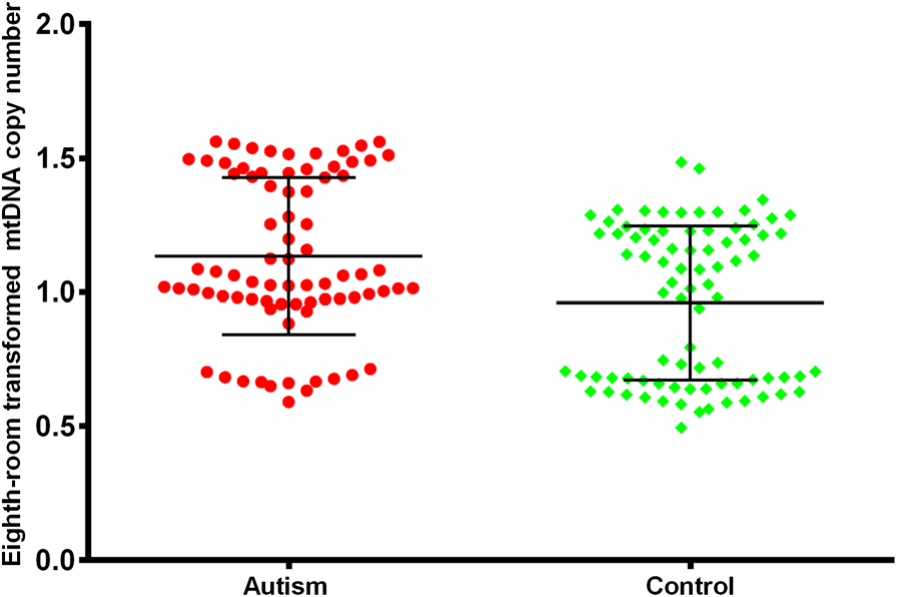
**Relative mtDNAcopy number of autistic patients and health controls.**

Linear regression analysis of both mtDNA and transformed mtDNA while controlling for age and sex showed an elevated mtDNA copy number in autism cases (Table 3). In the transformed mtDNA, mtDNA copy number is lower in controls compared to the autism cases (beta=−0.173, p=0.0003; Figure 1). Age and sex were not associated with mtDNA and transformed mtDNA. Stratification analysis by sex also showed the similar trend in males (beta=−0.172, p=0.0008). However, we did not observe significant association of mtDNA copy number with the disease in females (beta=−0.214, p=0.1056), although the effect size is slight larger. This may because the sample size is relative small and lack adequate statistical power to detect this association in females. We did not observe any significant interaction between the disease status and sex, and age.

**Table 3:** **Linear regression analysis of mtDNA and eighth-root transformed mtDNA and by sex in autism cases and controls**

	**mtDNA**			**Eighth-root transformed**		
**Parameter**	**Beta**	**SE**	**P**	**Beta**	**SE**	**P**
Overall						
Intercept	12.296	3.050	<.0001	1.248	0.103	<.0001
Control vs autism	−6.037	1.375	<.0001	−0.173	0.046	0.0003
Female vs Male	−0.317	1.904	0.8679	−0.0207	0.064	0.7469
Age	−0.062	0.056	0.2636	−0.0021	0.0019	0.2715
Male						
Intercept	11.976	2.906	<.0001	1.259	0.097	<.0001
Control vs autism	−5.849	1.490	0.0001	−0.172	0.050	0.0008
Age	−0.064	0.058	0.2735	−0.0028	0.0019	0.16
Female						
Intercept	11.763	8.332	0.172	0.9504	0.2782	0.0025
Control vs autism	−7.284	3.803	0.0685	−0.2144	0.1270	0.1056
Age	−0.032	0.193	0.8705	0.00535	0.0064	0.4143

**Figure 2:**
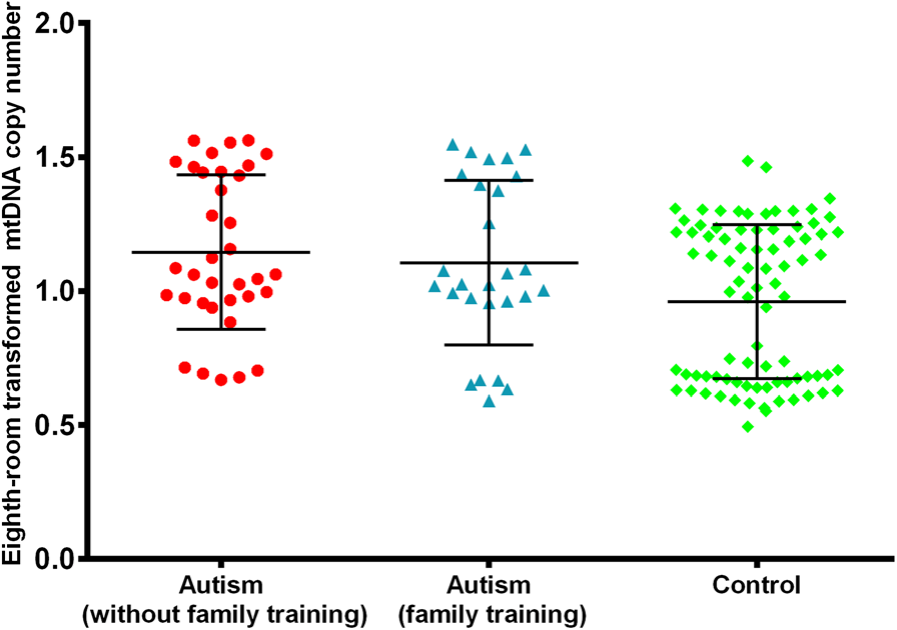
**MtDNA copy number in autism with and without family training compared with controls.** One-way analysis of variance (ANOVA) of the mean values was used to analyze the difference of mtDNA copy number among groups (F=6.042, P = 0.003) and the LSD test was used for multiple comparisons between any of the two groups. The differences of mtDNA copy number betweencontrol group and autism subgroup without family training, and autism subgroup withfamily trainingwere statistically significant (P = 0.002, 0.024, respectively). There is no significant difference in mtDNA copy number between patients with and without family training(p=0.592).

The associations between relative mtDNA copy number and clinical features in autism cases are shown in Table 4. No significant associations were observed between relative mtDNA copy number and clinical features (paternal age, maternal age, age of onset, illness of duration, CARS score and ABC score) in patients with childhood autism. Similarly, there was no difference of relative mtDNA copy number between patients with and without family training interaction (Figure 2).

**Table 4:** **Correlations between mtDNA copy number and clinical variables in childhood autism**

**Variables**	**Beta**	**P value**
Paternal age	0.009	0.297
Maternal age	0.002	0.778
Age of onset	−0.032	0.562
Illness of duration	−0.030	0.361
CARS Score	−0.002	0.873
ABC Score	−0.001	0.844

## Discussion

In this study, we measured the relative mitochondrial DNA copy number in peripheral blood cells using quantitative PCR technique in autism patients and healthy controls. Our study found significant difference in the relative mtDNA copy number between childhood autism and healthy controls, and an elevated mtDNA content level was observed in patients with autism.

Unlike the findings reported in previous studies that the mtDNA copy number was negatively correlative with age in healthy populations [34-36], this study showed no association between mtDNA copy number and age in case and control groups. This may due to the narrow age span of subjects included in our study (3–6 years), and lack of adequate statistical power to detect this association. In addition, no significant associations were observed between relative mtDNA copy number and paternal age, maternal age, age of onset, illness of duration, CARS score, ABC score, and family training status in childhood autism. This indicates that the mtDNA copy number has no direct impact on clinical features of autism.

Our finding is consistent with previous brain tissue-based study that showed an aberrant elevated mtDNA copy number in autism patients [20]. Gu et al. analyzed mtDNA copy number with the brain tissues in 9 autistic children and 9 controls, and they found increased copy numbers of three mitochondrial genes (ND1, ND4, CYTB) in autistic patients, which indicated higher mtDNA copy numbers in autism. However, this finding was failed to be replicated by a following postmortem study [26]. Tang et al. examined the of brain mtDNA in childhood subjects, including 8 ASD children and 7 controls and found that no mtDNA copy number are associated with ASD patients [26]. Similarly, Giulivi et al. evaluated mtDNA copy number in peripheral lymphocytes from 10 children with autism and 10 controls. They found that the mean mtDNA copy number in lymphocytes was not significantly different in overall association. However, 5 of 10 children with autism presented mtDNA over-replication compared with 95% CI of the value in control children [19]. A small sample size and the different tissues cells may account for this inconsistency in previous studies. We measured mtDNA content in peripheral lymphocytes from 161 subjects and found autistic children had higher copy numbers of mtDNA when compared with healthy children group. The increased mtDNA copy number may be caused by compensatory mtDNA over-replication or a disruption of mtDNA degradation, which indicated a dysfunctional state in mitochondria. Therefore, these findings showed mitochondrial dysfunction in children with autism and proved a previous finding that mitochondrial dysfunction may be a biological subtype of autism spectrum disorder [21].

The major strength of our study is the unique and highly homogenous patient subjects. ASD is a heterogeneous group of common developmental disorders including autism, Rett syndrome, Asperger’s syndrome and PDD-NOS. Our study is just restricted to childhood autism, excluding the patients with atypical autism and adult autism. Thus, any potential confounding effects yielded by different sub-diseases aetiology were eliminated, which may be efficient in this association study to evaluate the mtDNA status in childhood autism. Furthermore, we analyzed 161 children subjects in this study, which is much larger than the sample size in previous studies.

We have to note that there are several limitations in the present study. First, because the inherent limitation of case–control design, the finding may not be used to make causal inference on the association of elevated mtDNA copy number with the risk of autism; and potential population stratification was not controlled. In addition, while we analyzed mtDNA copy number in peripheral blood cells, brain tissues are considered as the standard target tissue for studying autism or other brain disorders. However, peripheral blood cells can be obtained in noninvasive method; and the mtDNA copy number in peripheral blood has been associated with that in brain tissues [37,38]. The mtDNA copy number in peripheral blood might serve as an alternative indicator for the mitochondrial function in brain tissues. Furthermore, the incomplete data on characteristics of autism and controls did not allow controlling more potential confounding.

## Conclusion

In this study we observed the mtDNA copy number in peripheral blood is significantly elevated in children with autism. Our finding supports the previous statement that there is mitochondrial dysfunction in patients with autism and indicates mitochondrial dysfunction may be a biological subtype of childhood autism. Additional research is needed to assess whether the association is replicable in the future.

## Abbreviations

mtDNA: mitochondrial DNA
ASD: Autism spectrum disorder
PDD-NOS: Pervasive developmental disorder-not otherwise specified
ATP: Adenosine triphosphate
ETC: Electron transport chain
ROS: Reactive oxygen species
DSM-IV: Diagnostic and statistical manual of mental disorders fourth edition
CARS: Childhood autism rating scale
ABC: Autism behavior checklist
PCR: Polymerase chain reaction
ND1: Nicotinamide Adenine Dinucleotide Hydrogen dehydrogenase gene 1
ND4: Nicotinamide Adenine Dinucleotide Hydrogen dehydrogenase gene 4
CYTB: Cytochrome-b encoded complex III subunit gene
